# Metformin Improves Endothelial Function and Reduces Blood Pressure in Diabetic Spontaneously Hypertensive Rats Independent from Glycemia Control: Comparison to Vildagliptin

**DOI:** 10.1038/s41598-017-11430-7

**Published:** 2017-09-08

**Authors:** Mahdi Hamidi Shishavan, Robert H. Henning, Azuwerus van Buiten, Maaike Goris, Leo E. Deelman, Hendrik Buikema

**Affiliations:** Department of Clinical Pharmacy and Pharmacology, University Medical Center Groningen, University of Groningen, Groningen, The Netherlands

## Abstract

Metformin confers vascular benefits beyond glycemia control, possibly via pleiotropic effects on endothelial function. In type-1-diabetes-mellitus (T1DM-)patients metformin improved flow-mediated dilation but also increased prostaglandin(PG)-F_2α_, a known endothelial-contracting factor. To explain this paradoxical finding we hypothesized that metformin increased endothelial-vasodilator mediators (e.g. NO and EDHF) to an even larger extent. Spontaneously-hypertensive-rats (SHR) display impaired endothelium-dependent relaxation (EDR) involving contractile PGs. EDR was studied in isolated SHR aortas and the involvement of PGs, NO and EDHF assessed. 12-week metformin 300 mg/kg/day improved EDR by up-regulation of NO and particularly EDHF; it also reduced blood pressure and increased plasma sulphide levels (a proxy for H_2_S, a possible mediator of EDHF). These effects persisted in SHR with streptozotocin (STZ)-induced T1DM. Vildagliptin (10 mg/kg/day), targeting the incretin axis by increasing GLP-1, also reduced blood pressure and improved EDR in SHR aortas, mainly via the inhibition of contractile PGs, but not in STZ-SHR. Neither metformin nor vildagliptin altered blood glucose or HbA_1c_. In conclusion, metformin reduced blood pressure and improved EDR in SHR aorta via up-regulation of NO and particularly EDHF, an effect that was independent from glycemia control and maintained during T1DM. A comparison to vildagliptin did not support effects of metformin mediated by GLP-1.

## Introduction

Metformin is an effective oral anti-diabetic drug, which decreases hepatic glucose production and increases peripheral glucose uptake in skeletal muscle. The UK Prospective Diabetes Study (UKPDS) established cardiovascular benefit and improved survival in metformin treatment compared with conventional treatment in overweight type-2 diabetes mellitus (T2DM) patients^1^. Given that the glucose-lowering effects of metformin, sulphonylurea and insulin in UKPDS were similar, it has been proposed that metformin confers additional vascular benefits beyond those of glycaemia control alone^2, 3^. DM often coexists with hypertension (HT)^4, 5^ and the results of large clinical studies suggest that the benefits of tight blood pressure control in T2DM patients are as important or may be even greater than the benefits of a more intensive glycemia control^6, 7^. Some clinical studies have reported a blood pressure lowering effect of metformin^8–10^ although others reported the lack of such effects^11, 12^.

Clinical studies reporting on metformin treatment associated with the improvement of endothelial function appear more consistent^13–15^. This is of interest because endothelial dysfunction is considered among the earliest vascular abnormalities induced by cardiovascular risk factors and a strong predictor of cardiovascular events in several patient groups^16^. In a study by Mather *et al*.^13^ in T2DM patients, metformin improved both insulin-resistance and acetylcholine-stimulated flow, with a strong statistical link between these variables. In another study, long-term metformin in T2DM subjects improved certain circulating markers of endothelial function independently from changes in HbA_1c_, insulin dose and weight, which may explain why it is associated with a decreased risk of cardiovascular disease in these patients^14^.

Metformin has also been investigated in type 1 (T1) DM patients, but only one study assessed its effects on endothelial function^17^. In that study, 6-months of metformin treatment, in addition to a basal-bolus regimen of insulin, in uncomplicated T1DM patients resulted in a significant improvement of flow-mediated dilation (FMD), a marker of systemic endothelial function. At the same time however, metformin increased prostaglandin (PG) F_2α_ in these patients which seems ‘paradoxical’ because PGF_2α_ is a marker of oxidative stress products, known to negatively affect endothelial function^17^. Together with PGH_2_ and thromboxane A_2_ (TxA_2_), PGF_2α_ is a potent vasoconstrictor prostanoid derived from endothelial cyclooxygenase (COX) and known to oppose endothelium-dependent relaxation (EDR)^18^. One possibility to explain the improved FMD response after metformin in face of increased (contractile) PGF_2α_ may be that metformin increased other endothelial vasodilator mediators such as nitric oxide (NO) and/or endothelium-derived hyperpolarizing factor (EDHF) to an even larger extent.

Spontaneously hypertensive rats (SHR) constitute a model of endothelial dysfunction involving increased production of endothelial COX-derived contracting factor(s) in a setting of HT^18, 19^. In the present study, streptozotocin (STZ) was used to induce DM in SHR to further investigate the treatment effects of metformin on endothelial dysfunction in hypertensive T1DM. Diabetic and non-diabetic SHR received metformin for 12 weeks or remained untreated after which blood pressure was assessed and the isolated aorta studied for different endothelial mediators involved in EDR. Interestingly, metformin was recently shown to increase glucagon-like peptide-1 (GLP-1) concentrations and GLP-1 receptor expression in mouse islets in a PPARα-dependent, AMPK-independent mechanism^20^, thus suggesting a role for the incretin axis in mediating some of the effects of metformin. To explore this, additional comparisons were made to SHR treated with vildagliptin. Vildagliptin is an anti-diabetic drug which targets the incretin axis by increasing GLP-1 via inhibition of the enzyme dipeptidyl-peptidase 4 (DPP-4)^21^. Vildagliptin has also been shown to improve endothelium-dependent vasodilation in T2DM patients^22^ but its effect on endothelial dysfunction in (hypertensive) T1DM has not been studied previously.

## Results

### Rat characteristics of SHR and STZ-SHR

**Figure 1:**
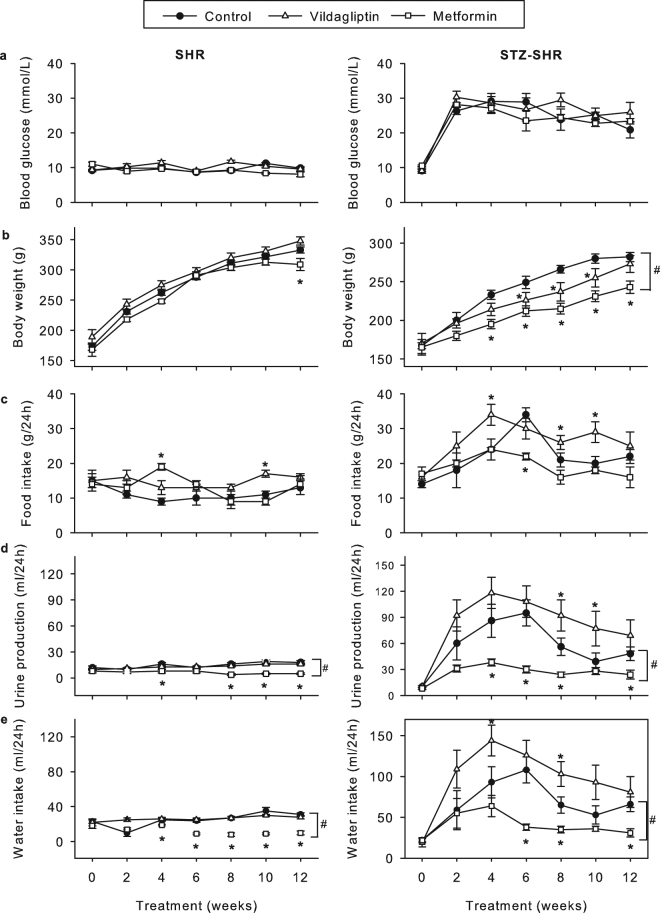
Effects of vildagliptin and metformin on SHR characteristics. Eight week old spontaneously hypertensive rats (SHR) rats were injected at t = 0 with streptozotocin (STZ, 60 mg/kg) or saline (non-STZ) to induce type-1 diabetes mellitus or not before they were treated for 12 weeks either with vildagliptin (10 mg/kg/day; open triangles) or metformin (300 mg/kg/day; open squares), or remained untreated (closed circles). Shown are the over time effects on (**A**) blood glucose, (**B**) body weight, (**C**) food intake, (**D**) urine production and (**E**) water intake. Data are mean ± SEM (n = 6–8 for each group). *Indicates p < 0.05 vs. untreated (STZ-)SHR at a specific time point; ^#^indicates p < 0.05 vs. untreated (STZ-)SHR for the whole curve.

Injection of SHR with STZ at the start of the study typically induced changes related to the development of DM. These included a significant increase in blood glucose, a reduced gain in body weight despite increased food intake, and increased urine production and water intake (Fig. 1). Twelve weeks after induction of T1DM at the end of the study period, STZ-SHR had increased blood HbA_1c_ and triglycerides, and showed early signs of development of end-organ damage. That is, relative liver and kidney weight were significantly increased compared to control SHR, as was urinary albumin excretion (Table 1). In contrast, systolic blood pressure (SBP) did not differ between SHR and STZ-SHR (Table 1).

**Table 1:** Overview of the study groups and rat characteristics at the end of the study period.

	SHR			STZ-SHR		
	Control (n = 8)	Vildagliptin (n = 8)	Metformin (n = 6)	Control (n = 7)	Vildagliptin (n = 7)	Metformin (n = 7)
body weight (g)	333 ± 5	348 ± 7	309 ± 10^#^	282 ± 6*	273 ± 11	243 ± 8^#^
food intake (g·day^−1^)	12.8 ± 1.8	15.5 ± 0.4	13.8 ± 2.9	22.1 ± 2.0*	24.6 ± 4.0	16.1 ± 2.7
water intake (ml·day^−1^)	31.4 ± 2.1	28.4 ± 1.5	10.0 ± 2.3^#^	66.3 ± 9.1*	80.6 ± 18.9	31.3 ± 5.0^#^
urine volume (ml·day^−1^)	17.5 ± 1.9	15.6 ± 1.2	4.5 ± 0.6^#^	48.3 ± 7.9*	68.9 ± 18.0	24.2 ± 5.4^#^
blood glucose (mmol l·L^−1^)	9.9 ± 0.3	9.6 ± 0.6	8.1 ± 0.7	20.9 ± 2.4*	25.9 ± 2.8	23.4 ± 0.8
HbA_1c_ (%)	3.71 ± 0.03	3.90 ± 0.07	3.78 ± 0.17	8.09 ± 0.34*	8.70 ± 0.55	7.31 ± 0.23
triglycerides (mmol·L^−1^)	0.80 ± 0.002	1.17 ± 0.29	0.95 ± 0.06	2.09 ± 0.30*	1.91 ± 0.32	1.90 ± 0.23
liver: body weight (mg·g^−1^)	34.9 ± 0.5	34.8 ± 0.8	34.8 ± 0.9	49.0 ± 1.2*	49.5 ± 1.1	48.0 ± 1.1
left kidney: body weight (mg·g^−1^)	3.85 ± 0.15	3.91 ± 0.30	4.17 ± 0.22	5.13 ± 0.31*	5.26 ± 0.14	4.99 ± 0.13
albumin excretion (mg·24 h^−1^)	9.5 ± 1.7	13.1 ± 2.9	25.5 ± 6.3^#^	28.4 ± 7.3*	23.8 ± 8.1	46.9 ± 6.7
systolic blood pressure (mmHg)	149 ± 6	119 ± 9^#^	112 ± 18^#^	134 ± 9	111 ± 7	92 ± 12^#^

Data are mean ± SEM. *p < 0.05 STZ-SHR vs. control SHR; ^#^p < 0.05 treatment vs. appropriate control.

### Effect of treatments on rat characteristics

Treatment with vildagliptin or metformin did not alter blood glucose (Fig. 1A), HbA_1c_ and triglycerides (Table 1), neither in control SHR nor in STZ-SHR. Vildagliptin tended to decrease the gain in body weight, and increase the production of urine and water intake in STZ rats during the treatment period, but at the end of the study period this was not significantly different from untreated STZ-SHR (Fig. 1). In contrast, metformin significantly reduced the gain in body weight, as well as the urine production and water intake in STZ rats. This effect was consistent over the whole study period and to some extent also visible in non-STZ rats (i.e. urine production and water intake; see Fig. 1). Metformin additionally reduced SBP both in SHR and STZ-SHR rats (Table 1). Blood pressure lowering effects were also observed after vildagliptin although statistically significant reduction in SBP was obtained in SHR only, but not STZ-SHR (Table 1). Finally, metformin raised urinary albumin excretion in STZ-SHR, albeit not significantly, while vildagliptin treatment did not affect this parameter (Table 1).

### Endothelium dependent relaxation in SHR and STZ-SHR

**Figure 2:**
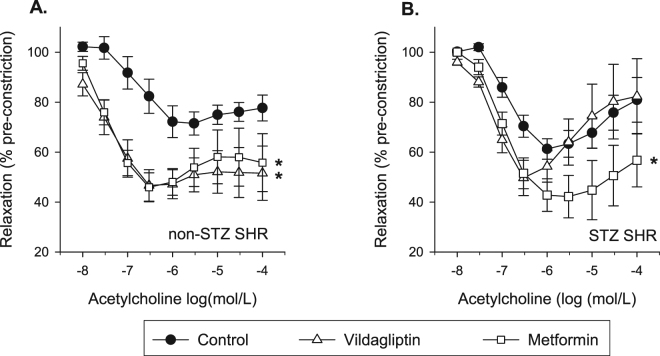
Effects of vildagliptin and metformin on endothelium-dependent relaxation in SHR aorta. Eight week old spontaneously hypertensive rats (SHR) rats were injected at t = 0 with streptozotocin (STZ-SHR, 60 mg/kg) or saline (SHR) to induce type-1 diabetes mellitus or not before they were treated for 12 weeks either with vildagliptin (10 mg/kg/day; open triangles) or metformin (300 mg/kg/day; open squares), or remained untreated (control; closed circles). Shown are the concentration-response curves of acetylcholine (ACh-)induced relaxation in isolated aorta preparations obtained at the end of the study period for A) SHR and B) STZ-SHR rats. Data are mean ± SEM (n = 6–8 for each group). *Indicates p < 0.05 vs. control for the whole curve (repeated measures ANOVA).

**Figure 3:**
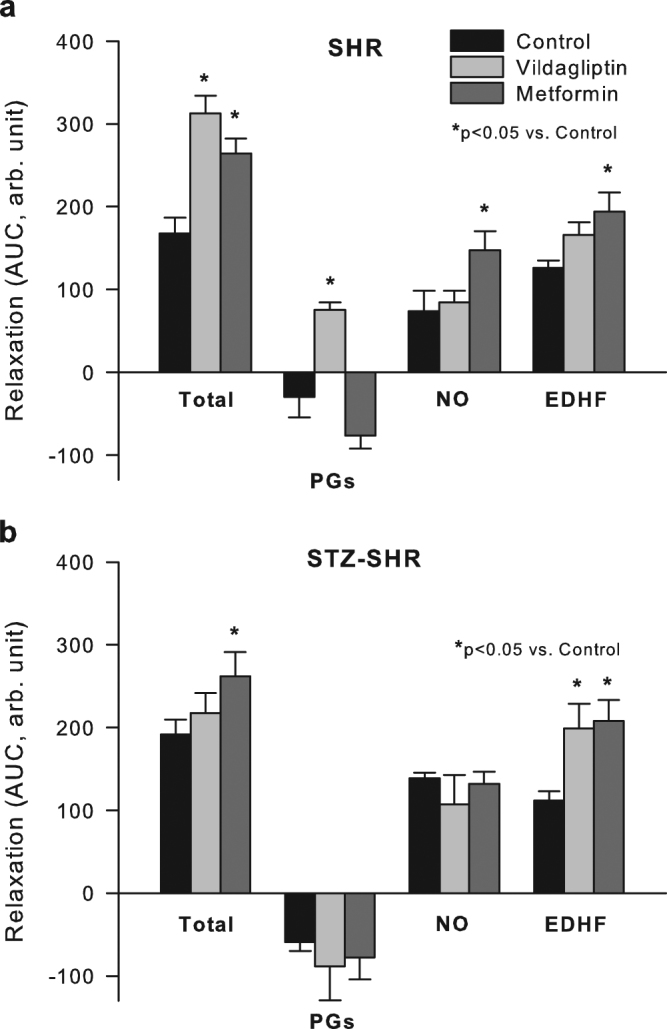
Effect of vildagliptin and metformin on endothelial mediators in SHR aorta. Acetylcholine-induced relaxation was studied in the absence or presence of indomethacin and/or LNMMA after which the area under the curve (AUC) was determined and used to calculate the contribution of prostaglandins (PGs, sensitive to COX-inhibition), nitric oxide (NO, sensitive to eNOS-inhibition) and EDHF (resistant to COX- plus eNOS-inhibition) to total relaxation (see also methods). Panel A and B shows the results for SHR and STZ-SHR, respectively. Data are mean ± SEM (n = 6–8 for each group). *Indicates p < 0.05 vs. control.

Full ACh-induced CR-curves in the absence of inhibitors are shown in Fig. 2. Hence, the relaxations were studied in aorta rings after pre-constriction with PE, the level of which did not statistically differ between the groups (i.e. neither in absolute terms nor when expressed as a percentage of the response to high potassium; Table 2). ACh typically induced a biphasic response showing relaxation at the lower concentrations and contraction at the higher concentrations^19^. This pattern - in particular the upstroke (Table 2) - appeared slightly more outspoken in STZ-SHR (Fig. 2B) compared to control SHR (Fig. 2A) but net relaxations did not significantly differ between both groups in terms EC_50_-values or maximal relaxation (Table 2), nor when the whole CR-curve (repeated measures ANOVA p = 0.47) or the AUC (Table 2, Fig. 3) was compared.

**Table 2:** Overview of curve characteristics for acetylcholine-induced relaxation in isolated rat aorta’s.

	SHR			STZ-SHR		
	Control	Vildagliptin	Metformin	Control	Vildagliptin	Metformin
PE pre-constriction						
absolute in µm	167 ± 3	155 ± 13	158 ± 9	163 ± 12	152 ± 12	213 ± 21
% high K^+^	89.1 ± 5.6	75.6 ± 6.0	84.8 ± 5.2	81.9 ± 8.0	87.4 ± 12.6	87.3 ± 10.1
ACh-induced relaxation						
EC_50_-value (log M)	−6.67 ± 0.24	−7.46 ± 0.21^#^	−7.53 ± 0.11^#^	−6.82 ± 0.05	−7.03 ± 0.14	−7.02 ± 0.06
constriction remaining at maximal relaxation (% PE)	71.5 ± 4.6	47.1 ± 6.7^#^	45.9 ± 5.8^#^	61.3 ± 4.1	50.7 ± 8.7	42.2 ± 6.5
Upstroke after maximal relaxation (∆ % PE)	6.2 ± 3.8	4.4 ± 6.2	9.9 ± 15.0	19.6 ± 4.4*	32.7 ± 22.6	14.6 ± 3.3
AUC (arbitrary units)	170 ± 19	313 ± 21^#^	264 ± 18^#^	192 ± 18	217 ± 24	262 ± 28^#^

Data are mean ± SEM and correspond to the data in Fig. 2. *p < 0.05 STZ-SHR vs. control SHR; ^#^p < 0.05 treatment vs. appropriate control.

Consistent with literature^19^, inhibition of COX-derived vasoactive eicosanoids by pre-incubation with indomethacin (INDO) prevented the ‘upstroke’ at the higher concentrations of ACh (data not shown as a CR-curve), thereby increasing net relaxation by ~25% (AUC from 181 ± 13 to 225 ± 12 arb. unit, paired t-test p = 0.007) for untreated SHR and STZ-SHR combined. Induction of T1DM tended to increase the involvement of contractile PGs (from −30 ± 25 arb. unit in SHR to −59 ± 10 arb. unit in STZ-SHR) but this was not significant (t-test p = 0.299). At the same time T1DM evoked an up-regulation of opposing NO-influence (from 74 ± 25 arb. unit in SHR vs. 139 ± 7 arb. unit in STZ-SHR; t-test p = 0.030), while the involvement of EDHF remained unaltered (see also Fig. 3).

### Effect of treatments on endothelium dependent relaxation

In control SHR, treatment with vildagliptin increased the sensitivity and maximal relaxation to ACh and profoundly increased the overall relaxation response as compared to those untreated (Fig. 2, Table 2). This increased response was largely abolished when relaxations were studied in the presence of INDO, which suggests that the enhancing effect was mainly attributable to an increased involvement of COX-derived dilatory PGs after vildagliptin (Fig. 3A). Similarly, metformin treatment also increased the sensitivity and maximal relaxation to ACh (Table 2) and the net relaxation to ACh (Fig. 2, Table 2). Contrary to vildagliptin treated rats however, ACh-induced relaxation in metformin treated animals increased in the presence of INDO (Fig. 3A), suggesting that metformin did not affect contractile PGs. Instead, the enhancing effect of metformin treatment on net ACh-induced relaxation in SHR was fully attributable to an increased NO- and EDHF-contribution (Fig. 3A).

The above enhancing effects of metformin treatment on EDR seemed largely maintained when T1DM was induced. That is, the profile of ACh-induced relaxation was not significantly altered when STZ was used to induce T1DM in SHR (compare panels A and B in Fig. 3). In contrast, the enhancing effect of vildagliptin treatment on ACh-induced relaxation as seen in control SHR was dramatically overcome in T1DM rats, resulting in vildagliptin no longer improving net ACh-induced relaxation (Fig. 2, Table 2). This appeared most crucially due to the loss of vildagliptin treatment to alter the involvement of contractile PGs in STZ-SHR (Fig. 3B). Although EDHF was significantly up-regulated in STZ rats treated with vildagliptin, this was no longer sufficient to improve net ACh-induced relaxation in SHR with T1DM (Fig. 3B). A summary of the directional changes induced by the treatments is also provided in Table 3.

**Table 3:** Summary of directional changes in endothelial mediators in acetylcholine-induced relaxation in SHR aorta: effect of treatments.

Effect of treatment in	Total relaxation	PGs (sensitive to INDO)	NO (sensitive to LNMMA)	EDHF (INDO + LNMMA resistant)
SHR				
vildagliptin	**↑↑**	**↑↑**	**≈**	**≈↑**
metformin	**↑↑**	**≈↓**	**↑↑**	**↑↑**
STZ-SHR				
vildagliptin	**≈**	**≈↓**	**≈↓**	**↑↑**
metformin	**↑↑**	**≈**	**≈**	**↑↑**

Symbols indicate directional changes induced by treatments compared to untreated SHR and untreated STZ-SHR. Double symbols ↓↓ and ↑↑ indicate a significant decrease and increase, respectively.

### Linear regression analysis of endothelium dependent relaxation, systolic blood pressure and plasma sulphide levels

**Figure 4:**
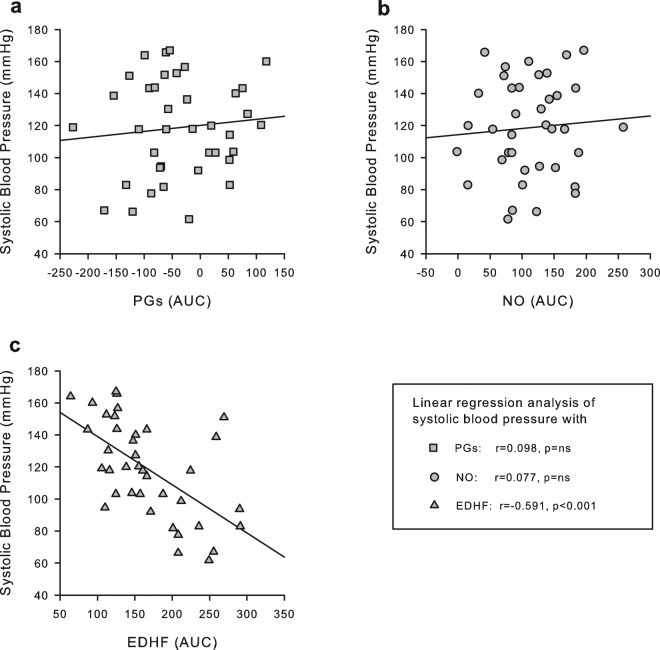
Linear regression analysis of endothelial mediators of aorta EDR with SBP. Shown are scatterplots for the contribution of, respectively, (**A**) prostaglandins (PGs), (**B**) nitric oxide (NO) and (**C**) EDHF with systolic blood pressure (SBP). Results of linear regression analysis are as indicated, with EDHF being the only mediator significantly correlated with SBP.

**Figure 5:**
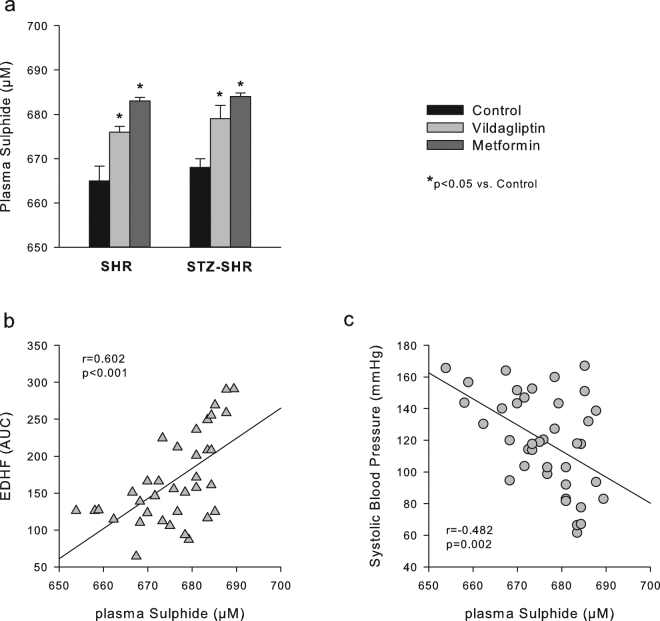
Plasma sulphide levels and its correlation with EDHF and SBP. Shown are (**A**) the average plasma sulphide levels (as a proxy of H_2_S) in treated and untreated SHR and STZ-SHR, and scatterplots of individual plasma sulphide levels with (**B**) EDHF contribution in aorta endothelium dependent relaxation and with (**C**) systolic blood pressure. Results of linear regression analysis are as indicated.

The only endothelial mediator involved in EDR significantly correlating with blood pressure was EDHF, such that a higher EDHF linearly correlated with a lower blood pressure (Fig. 4). As H_2_S has recently been identified as a new EDHF^23, 24^, we additionally assessed plasma sulphide levels (as a proxy of H_2_S) and also analyzed these for correlations with EDHF and SBP. Sulphide levels were significantly increased after vildagliptin and in particular after metformin (Fig. 5A). Interestingly, sulphide levels positively correlated with EDHF and inversely with SBP (Fig. 5B,C).

## Discussion

The main finding of the present study is that treatment of SHR with metformin and vildagliptin (1) improved endothelial function and reduced blood pressure (2) independently of glycemia control, (3) yet via differential mechanisms as it appears. Metformin improved aorta EDR by up-regulation of NO and in particular EDHF without attenuating the involvement of contractile PGs, an effect that also persisted in SHR with STZ-induced T1DM. In contrast, improved EDR after vildagliptin treatment in SHR was mostly attributable to the inhibition of contractile PGs, an effect however that did not persist in STZ-SHR. The findings implicate that (1) metformin and vildagliptin both have a pleiotropic potential to improve endothelial function and reduce blood pressure, but also (2) highlight the way their effects are achieved via different mechanisms, and (3) the effectiveness of vildagliptin herein being dependent on the specific conditions concerned. These findings seem of relevance to the treatment of HT-patients with DM since improvement of endothelial function and blood pressure reduction - whether it be achieved primary or secondary - are considered important targets for pharmacotherapy to prevent the high incidence cardiovascular morbidity and mortality in these conditions.

SHR is a well-studied model of endothelial dysfunction involving increased contractile PGs^18, 19^. To our knowledge only few studies have previously investigated the treatment effects of metformin in SHR but none of these assessed its effect on EDR. Moreover, the effects of vildagliptin so far have not been investigated in SHR at all. Verma *et al*.^25^ in an early study observed that treatment of five-week old SHR with (500 mg/kg/day) metformin for 2 weeks decreased plasma insulin levels and attenuated the increase SBP without affecting the plasma glucose levels. Blood pressure reductions have also been reported in some clinical studies such as in non-diabetic, obese, hypertensive women receiving metformin^10^ and with vildagliptin in T2DM patients inadequately controlled with metformin^26^. However, the findings in humans are not consistent and could not be dissociated from glucose lowering effects. In the present study, metformin and vildagliptin did not affect blood glucose levels and HbA_1c_ in our (STZ-)SHR, thus implying that the observed treatment effects in the present study were independent of (hyper)-glycemia control.

Metformin treatment tended to increase contractile PGs (albeit not significant) yet up-regulated NO and in particular EDHF to a significantly larger extent, thereby profoundly improving net EDR. Since this effect persisted in STZ-SHR it may also provide an explanation for the ‘paradoxidal’ findings by Pitocco *et al*.^17^ who observed improved FMD response in face of increased (contractile) PGF_2α_ after metformin in T1DM patients. Recently, Tsai *et al*.^27^ reported that metformin may exert an anti-hypertensive effect in SHR by restoration of the ADMA-NO pathway. Increased NO bioactivity after metformin is also in agreement with the results of previous studies by Davis *et al*.^28^ which suggested that metformin improves endothelial vascular function in DM by increasing AMPK-dependent, hsp90-mediated eNOS activation.

In addition to NO, metformin has been shown to raise hydrogen sulfide (H_2_S) tissue concentration in various mouse organs^29^. This seems of relevance because H_2_S has been identified as a new EDHF that sulfhydrates potassium channels^23, 24^ and functions a vasculoprotective factor^30^. Hence, up-regulation by metformin of H_2_S as an EDHF could account for the increased ACh-induced relaxation that was resistant to COX- and LNMMA-inhibition, and which positively correlated with plasma sulphide levels (as a proxy of plasma H_2_S levels) in the present study. Furthermore, administration of exogenous H_2_S has been shown to reduce blood pressure in STZ-SHR^31^. An up-regulation of H_2_S as an EDHF contributing to the anti-hypertensive effect of metformin - as suggested by the inverse correlations of EDHF and plasma sulphide levels with SBP in the present study - could be in agreement herewith.

Finally, some studies have reported the ability of metformin to reduce vascular production of vasoconstrictor PGs^32, 33^, which is in contrast with our present findings. However, in these studies different rat models were used, i.e. fructose-overloaded rat of metabolic syndrome, aged OLETF rat model of T2DM. Furthermore, the reduced vascular production of vasoconstrictor PGs may have been secondary to the reduction in hyperglycemia after metformin in those studies, but not in the present. Overall, there seems limited and/or controversial evidence for a (pleiotropic) action of metformin to improve EDR via the inhibition of COX-derived contractile PGs (independent of glycemia control).

Although the effect of vildagliptin has not been investigated previously in SHR, our present findings seem in line with other studies suggesting that GLP-1 elevating or GLP-1 receptor activating agents may restore endothelial function and reduce SBP in SHR^34, 35^. Liu *et al*.^36^ previously reported sitagliptin to improve EDR in SHR renal arteries via a GLP-1-dependent mechanism involving the restoration of NO bioavailability. Vildagliptin also improved EDR in the present study, albeit not via increased NO as it appeared. Nevertheless, study differences in, amongst others, the vessel type investigated (e.g. aorta vs. renal artery), experimental conditions employed (e.g. use of INDO or not) and the way endothelial mediators were assessed (e.g. functional measurement of EDR sensitive to eNOS-inhibition vs. a direct measurement of NO-production) are likely contributors to such apparent discrepancies, although without necessarily excluding each other. For that matter, our results do seem in line with another recent study of the aforementioned group in which they reported sitagliptin to restore endothelial function in SHR renal arteries and in angiotensin II-infusion induced hypertensive C57BL/6 mouse aorta via the up-regulation of uncoupling protein 2 (UPC2) in a GLP-1-dependent pathway, leading to a reduced production of reactive oxygen species and subsequent reduction of COX-2 expression and COX-2-derived endothelium-derived contractile factor(s) (EDCFs)^36^. Although we did not study UPC2 and COX-expression in the present study, our finding that vildagliptin improved EDR via the inhibition of COX-derived contractile PGs opposing net relaxation is in agreement herewith.

As distinct from metformin, the enhancing effect of vildagliptin on EDR was abolished when SHR had received STZ to induce T1DM. In seeming conjunction herewith, also the anti-hypertensive effect of vildagliptin - although still present to some extent - no longer reached statistical significance in STZ-SHR, thus indicating that presence of *per se* T1DM importantly modulated the vascular/hemodynamic effects of vildagliptin. A lack of improvement of EDR after vildagliptin in a setting of endothelial dysfunction in DM is consistent with our previous findings in Zucker diabetic fatty (ZDF) rats, a model of T2DM that is also characterized by impaired aorta EDR due to COX-derived contracting factors albeit in a normotensive setting^37^. In that study, vildagliptin similarly “failed” to inhibit the opposing role of COX-derived contractile PGs and to improve EDR in ZDF rat aorta^37^. Notably however, both in that study in T2DM ZDF rats^37^ as well in the present study in T1DM STZ-SHR, triglycerides were increased. And in both studies vildagliptin treatment did not improve EDR, nor the increased triglycerides levels in DM. Interestingly in this context is the study by Nathanson *et al*.^38^ who *ex vivo* investigated the effect of exenatide - an GLP-1 analogue - on EDR in rat femoral artery. These authors performed a short term pre-incubation study of this conduit artery with intralipid to induce triglyceride-induced endothelial dysfunction and found that exenatide did not restore EDR^38^, which is in general consistency with our present findings (i.e. from a treatment point of view). Additionally interesting is the fact that these authors noticed a concomitant “paradoxical” increase in eNOS activity following intralipid pre-incubation, but which apparently was insufficient to compensate and restore or improve EDR. In the present study, induction of TIDM also raised the NO-contribution (in STZ-SHR vs, SHR) in EDR, albeit not significant, but possibly as a counteractive mechanism (i.e. from a physiologic point of view). Nevertheless, what remains is the discrepancy between vildagliptin treatment effects on EDR in different pathophysiological conditions.

The effects of metformin and vildagliptin on EDR in (STZ-)SHR aorta in the present study may be considered pleiotropic, i.e. taking into account the lack of effect on glycemia control, but not independent of blood pressure reduction. That is, given the fact that HT is a *per se* risk factor for endothelial dysfunction and *vice versa*, the present study does not dissociate between a blood pressure lowering effect facilitating the improvement of endothelial function or the other way around. Given our present findings however, we *tend to interpret* the blood pressure lowering effects more as a result of the treatment’s effects on vascular endothelial function rather than *vice versa*. Particularly the observed intercorrelationships in the present study between vascular EDHF, systemic sulphide levels and arterial SBP in the overall context of literature in our view add to this opinion. Nevertheless, we did not investigate whether the EDR-part resistant to combined eNOS- and COX-inhibition (denoted as EDHF) was actually mediated by H_2_S, nor can we exclude that metformin’s treatment tendency to reduce body weight played some role herein.

High blood glucose is associated with oxidative stress which in turn may act as a trigger of H_2_S production^39^. Literature suggests a relation between H_2_S and glucose level in diabetic patients as well as in diabetic rodents although the findings seem not unambiguous. Some investigators reported enhanced plasma H_2_S levels to be associated with fasting blood glucose in T2DM patients^40^, while others make notion of reduced plasma H_2_S levels in diabetic patients and/or STZ-induced diabetic rats^41, 42^. Here we found no differences in plasma H_2_S levels between SHR and STZ-SHR rats. This may be in line with findings of Yusuf *et al*.^43^ reporting similar plasma H_2_S levels in STZ-induced diabetic and non-diabetic Sprague Dawley rats. At the same time in that study, tissue H_2_S formation in the pancreas and liver was upregulated. This led the authors to suggest enhanced formation of H_2_S likely to be a ‘local’ tissue response to diabetes, which is not reflected in increased circulating levels^43^. Interestingly, metformin did raise circulating H_2_S levels both in our SHR and STZ-SHR rats and improved endothelial function, particularly by increasing EDHF. This may point at a ‘local’ tissue action of metformin independent of glycemic control but not necessarily independent of insulin sensitivity. Hence, a reduced insulin resistance or increased insulin sensitivity after metformin can also increase the sensitivity of the vasculature for the relaxants by stimulating multiple pathways including NO and EDHF^44–46^.

In summary, the results highlight that metformin and vildagliptin differed in their pleiotropic potential to improve endothelial function. I.e. both compounds improved net EDR in SHR yet via the mobilization of different endothelial mediators. It seems that inhibition of contractile PGs in SHR aorta may be an effect more related to GLP-1 since it was found in vildagliptin treatment only, but not metformin. On the other hand, both vildagliptin and particularly metformin increased (plasma sulphide and) EDHF. No study has previously investigated the chronic effects of gliptins on EDHF, and only one study^47^ investigated the acute effects of alogliptin in C57BL/6 mice aorta. That study concluded that acute DPP-4 inhibition modulates vascular tone through GLP-1 independent pathways that are NO and EDHF dependent^47^. Hence, when compared to vildagliptin the results of our study also provide little or no support that metformin exerted its treatment effects via GLP-1. Interestingly, gliptins are increasingly used as an add-on to metformin in T2DM patients when metabolic control is not achieved^48^. Our present findings imply that metformin and vildagliptin improve endothelial function via different mechanisms but which might be complementary when given together. Whether this is the case and, if so, whether it contributes to a better organ protection as seen in experimental studies after combined vildagliptin plus metformin treatment^49^ may be subject of future investigation.

## Methods

### Animals and study design

The protocols for animal care and use were in accordance with EU Directive 2010/63/EU for animal experiments and approved by the Committee for Animal Experiments of the University of Groningen (Permit Number: DEC6032E). Studies were conducted using male SHR rats (n = 43) obtained from Charles River (France). Animals were housed group-wise in standard cages and maintained on a 12:12-hour light:dark cycle with free access to normal standard rat chow (Hope Farms B.V., Woerden, Netherlands) and normal tap drinking water throughout the study.

After one-week acclimatization when animals were at 7 weeks of age (150–200 g), a blood sample was drawn from the tail vein. After assessment of baseline parameters rats were randomly injected either with saline or 60 mg/kg i.p. STZ to induce T1DM^50^. This was followed one week thereafter by the determination of blood glucose (after 3 hour fasting) based on which STZ and non-STZ rats were stratified to treatment with 300 mg/kg/day metformin^51^, 10 mg/kg/day vildagliptin^52^ or no treatment. Starting at 8 weeks of age, treatments were initiated and maintained until sacrifice at 20 weeks of age. Treatments were provided via the drinking water and the amount of drug adjusted weekly according to body weight and water intake. When required, individual rats were provided an insulin implant (Linplant, Linshin Inc, Canada) to prevent excessive loss of body weight and maintain 3 hour fasting blood glucose levels within a target range of 16–28 mmol∙L^−1^ in accord with Lim *et al*.^53^. Since blood samples were obtained during mild anesthesia (2% isoflurane in oxygen) and immediately assessed for ‘bed-side’ glucose levels, subcutaneous placement of the insulin implant was performed during anesthesia at the same time. In the end, 3/7 STZ-SHR, 4/7 STZ-vildagliptin and 3/7 STZ-metformin rats were provided with a subcutaneous placement of the insulin implant.

Throughout the study period, 24 h urine production and water intake was monitored weekly (metabolic cages), and a blood sample from the tail vein for blood glucose was collected every 2 weeks. The arterial blood pressure was determined indirectly by the tail-cuff method at the end of the study period. After twelve weeks of treatment (i.e. when animals were 20 weeks of age), rats were anesthetized (2.5% isoflurane in oxygen) and the kidney and liver removed and weighed. The aorta was also removed and prepared for studies of EDR in organ bath experiments.

### Aorta studies of endothelium dependent relaxation

The thoracic descending aorta was excised and placed in a Krebs bicarbonate solution of the following composition (mmol∙L^−1^): NaCl, 120.4; KCl, 5.9; CaCl_2_, 2.5; MgCl_2_, 1.2; NaH_2_PO_4_, 1.2; glucose, 11.5; NaHCO_3_, 25.0; continuously aerated with 95% O_2_ and 5% CO_2_. The vessel was cleaned of adhering fat tissue and rings of 2 mm in length were cut with a sharp razor blade, with care not to touch the luminal surface. Rings were mounted between two stirrups in organ baths filled with 15 ml of Krebs solution. One stirrup was anchored inside the organ bath and the other connected to a displacement transducer to determine isotonic changes, as described previously^54^. Rings were subjected to 14 mN and allowed to stabilize for 60 min before they were primed and checked for viability by evoking a contraction with 60 mmol∙L^−1^ KCl twice. After washout and renewed stabilization, rings were pre-contracted with 1 µmol∙L^−1^ phenylephrine (PE) followed by the determination of the dilatory response to the endothelium-dependent vasodilator acetylcholine (ACh: 10 nmol∙L^−1^ to 100 µmol∙L^−1^) both in the absence (i.e. vehicle) and the presence of either 10 µmol∙L^−1^ indomethacin (INDO) alone (i.e. to inhibit COX-derived vasoactive eicosanoids), or in combination with 100 mmol∙L^−1^ N^G^-mono-methyl-L-arginine (L-NMMA, i.e. to additionally inhibit NO-synthase).

### Clinical chemistry

Blood glucose was determined using an Accu-Check Aviva monitor (Roche Diagnostics, Almere, the Netherlands). HbA_1c_ and urinary albumin were determined using a DCA Vantage Analyzer (Siemens Healthcare Diagnostics Inc., Deerfield, IL), and plasma triglycerides on a multitest analyzer system (Roche Modular; F. Hoffmann-La Roche Ltd., Basel, Switzerland). For assessment of plasma sulphide (as a proxy of H_2_S), sulphide antioxidant buffer (390 mmol∙L^−1^ sodium salicylate, 9.2 mmol∙L^−1^ ascorbic acid, 531 mmol∙L^−1^ NaOH) was prepared and added to blood samples obtained from the rats in a 1:1 ratio. An sulphide electrode was immersed into the mixture and the electrode potential was monitored and the stabilized mV reading recorded. The sulphide ion concentration of the blood was read using electrode standardization curve prepared according to the manufacturer’s guide (Lazar Research Laboratories, Inc. Los Angeles, CA, 90036 USA).

### Solutions and drugs

Compounds for Krebs solution and aorta studies were obtained from Merck (Darmstadt, Germany) and from Sigma-Aldrich (St. Louis, MO, USA). Metformin (Metformine HCl, Pharmachemie BV) and vildagliptin (Galvus, Novartis) were obtained from the Hospital Pharmacy at the University Medical Center Groningen, the Netherlands).

### Calculations and data analysis

Vasodilator responses to ACh were expressed as a percentage of PE-induced pre-contraction before individual CR-curves were generated and analyzed for curve characteristics. Because of the biphasic nature of the response only the first part of the CR-curve up to where relaxations to ACh became maximal was considered for determination of the EC_50_-value (SigmaPlot, Systat Inc., San Jose, CA). To further characterize the upward stroke at the higher concentrations of ACh the increase from the minimal contraction level remaining after ACh and the maximal contraction level remaining after addition of the higher concentrations of ACh was calculated. Finally the Area Under each individual Curve (AUC, in arbitrary units) was determined (SigmaPlot, Systat Inc., San Jose, CA) as a measure of the total response. The AUC was used to represent the individual response-size in a given condition, and where appropriate, to calculate the individual difference in response-size between different conditions (i.e. difference response-size in the presence of saline vs. INDO vs. INDO plus LNMMA for ACh). The AUC was also used to calculate and present the average response-size per group, and for subsequent analysis of differences in response-size among experimental groups. For that matter, the INDO-sensitive part of the response was regarded to be mediated by COX-derived PGs, the LNMMA-sensitive part to be mediated by NO, and the INDO + LNMMA-resistant part to be mediated by EDHF^54, 55^.

Unless stated otherwise, all data are presented as mean ± SEM; *n* values represent the number of investigated rats or valid observations obtained for a given parameter. IBM SPSS Statistics for Windows, Version 20.0 (Armonk, NY: IBM Corp.) was used for statistical analysis. Full CR-curves were also compared using repeated measurements ANOVA. Student’s unpaired t-test was used to compare control SHR with STZ-SHR. One-way ANOVA followed by LSD post-hoc testing for multiple comparisons was used to test the effect of treatment in control SHR or in STZ-SHR. Linear regression analysis was performed to further analyze possible relationships between mediators of EDR and SBP. P < 0.05 values (two-tailed) were considered statistically significant.

## References

[1] UK Prospective Diabetes Study (UKPDS) Group (1998). Effect of intensive blood-glucose control with metformin on complications in overweight patients with Type 2 diabetes (UKPDS 34). Lancet.

[2] Boyle JG, Salt IP, McKay GA (2010). Metformin action of AMP-activated protein kinase: a translational research approach to understanding a potential new therapeutic target. Diabet Med.

[3] Rena G, Pearson ER, Sakamoto K (2012). Molecular action and pharmacogenetics of metformin: current understanding of an old drug. Diabetes Management.

[4] Epstein M, Sowers JR (1992). Diabetes mellitus and hypertension. J Hypertension.

[5] Sowers JR, Epstein M, Frohlich EDD (2001). hypertension, and cardiovascular disease. An update. Hypertension.

[6] Zanella MT, Kohlman O, Ribeiro AB (2011). Treatment of obesity, hypertension and diabetes syndrome. Hypertension.

[7] Mogensen CE (1998). Combined high blood pressure and glucose in type 2 diabetes: double jeopardy: British trail shows clear effect of treatment, especially blood pressure reduction. BMJ.

[8] Landin-Wilhelmsen K (1992). Metformin and blood pressure. J Clin Pharm Ther.

[9] Chan JC, Tomlinson B, Critchley JA, Cockram CS, Walden RJ (1993). Metabolic and hemodynamic effects of metformin and glibenclamide in normotensive NIDDM patients. Diabetes Care.

[10] Giugliano D (1993). Metformin improves glucose, lipid metabolism, and reduces blood pressure in hypertensive, obese women. Diabetes Care.

[11] He H (2012). Metformin-based treatment for obesity-related hypertension: a randomized, double-blind, placebo-controlled trial. J Hypertens.

[12] Snorgaard O, Køber L, Carlsen J (1997). The effect of metformin on blood pressure and metabolism in nondiabetic hypertensive patients. J Intern Med.

[13] Mather KJ, Verma S, Anderson TJ (2001). Improved endothelial function with metformin in type 2 diabetes mellitus. J Am Coll Cardiol.

[14] de Jager J (2014). Long-term effects of metformin on endothelial function in type 2 diabetes: a randomized controlled trial. J Intern Med.

[15] Wu S, Li X, Zhang H (2014). Effects of metformin on endothelial function in type 2 diabetes. Exp Ther Med.

[16] Brunner H (2005). Working Group on Endothelins and Endothelial Factors of the European Society of Hypertension. Endothelial function and dysfunction. Part II: Association with cardiovascular risk factors and diseases. A statement by the Working Group on Endothelins and Endothelial Factors of the European Society of Hypertension. J Hypertens.

[17] Pitocco D (2013). Metformin improves endothelial function in type 1 diabetic subjects: a pilot, placebo-controlled randomized study. Diabetes Obes Metab.

[18] Qian H, Luo N, Chi Y (2012). Aging-shifted prostaglandin profile in endothelium as a factor in cardiovascular disorders. J Aging Res.

[19] Félétou M, Verbeuren TJ, Vanhoutte PM (2009). Endothelium-dependent contractions in SHR: a tale of prostanoid TP and IP receptors. Br J Pharmacol.

[20] Maida A, Lamont BJ, Cao X, Drucker DJ (2011). Metformin regulates the incretin receptor axis via a pathway dependent on peroxisome proliferator-activated receptor-α in mice. Diabetologia.

[21] Drucker DJ, Nauck MA (2006). The incretin system: glucagon-like peptide-1 receptor agonists and dipeptidyl peptidase-4inhibitors in type 2 diabetes. Lancet.

[22] van Poppel PC, Netea MG, Smits P, Tack CJ (2011). Vildagliptin improves endothelium-dependent vasodilatation in type 2 diabetes. Diabetes Care.

[23] Mustafa AK (2011). Hydrogen sulfide as endothelium-derived hyperpolarizing factor sulfhydrates potassium channels. Circ Res.

[24] Tang G (2013). H_2_S Is an Endothelium-Derived Hyperpolarizing Factor. Antioxid Redox Signal.

[25] Verma S, Bhanot S, McNeill JH (1994). Metformin decreases plasma insulin levels and systolic blood pressure in spontaneously hypertensive rats. Am J Physiol.

[26] Bosi E, Camisasca RP, Collober C, Rochotte E, Garber AJ (2007). Effects of vildagliptin on glucose control over 24 weeks in patients with type 2 diabetes inadequately controlled with metformin. Diabetes Care.

[27] Tsai CM, Kuo HC, Hsu CN, Huang LT, Tain YL (2014). Metformin reduces asymmetric dimethylarginine and prevents hypertension in spontaneously hypertensive rats. Transl Res.

[28] Davis BJ, Xie Z, Viollet B, Zou MH (2006). Activation of the AMP-activated kinase by antidiabetes drug metformin stimulates nitric oxide synthesis *in vivo* by promoting the association of heat shock protein 90 and endothelial nitric oxide synthase. Diabetes.

[29] Willinski B, Willinski J, Somogyi E, Piotrowska J, Opoka W (2013). Metformin raises hydrogen sulfide tissue concentrations in various mouse organs. Pharmacol Rep.

[30] Streeter E, Ng HH, Hart JL (2013). Hydrogen sulfide as a vasculoprotective factor. Med Gas Res.

[31] Ahmah FD (2012). Exogenous hydrogen sulfide (H_2_S) reduces blood pressure and prevents the progression of diabetic nephropathy in spontaneously hypertensive rat. Ren Fail.

[32] Puyó AM (2012). Metformin reduces vascular production of vasoconstrictor prostanoids in fructose overloaded rats. Auton Autacoid Pharmacol.

[33] Matsumoto T (2008). Metformin normalizes endothelial function by suppressing vasoconstrictor prostanoids in mesenteric arteries from OLETF rats, a model of type 2 diabetes. Am J Physiol Heart Circ Physiol.

[34] Pacheco BP (2011). Dipeptidyl peptidase IV inhibition attenuates blood pressure rising in young spontaneously hypertensive rats. J Hypertens.

[35] Liu L (2012). Dipeptidyl peptidase 4 inhibitor sitagliptin protects endothelial function in hypertension through a glucagon-like peptide 1-dependent mechanism. Hypertension.

[36] Liu L (2014). Uncoupling Protein-2 Mediates DPP-4 Inhibitor-induced restoration of endothelial function in hypertension through reducing oxidative stress. Antioxid Redox Signal.

[37] Wang Y (2012). Attenuation of renovascular damage in Zucker diabetic fatty rat by NWT-03, an egg protein hydrolysate with ACE- and DPP4-inhibitory activity. PLoS One.

[38] Nathanson D, Erdogdu O, Pernow J, Zhang Q, Nyström T (2009). Endothelial dysfunction induced by triglycerides is not restored by exenatide in rat conduit arteries *ex vivo*. Regul Pept.

[39] Pichette, Y., Gagnon, J. Implications of hydrogen sulfide in glucose regulation: how can H_2_S alter glucose homeostasis through metabolic hormones. *Oxid Med Cell Longev* 3285074 (2016). 10.1155/2016/3285074 PMC4958482 27478532

[40] Saha P (2015). Enhanced plasma H_2_S levels associated with fasting blood glucose in type-2 diabetes mellitus. AJMS.

[41] Jain SK (2010). Low levels of hydrogen sulfide in the blood of diabetes patients and streptozotocin-treated rats causes vascular inflammation?. Antioxid Redox Signal.

[42] Dutta M (2014). Evaluation of plasma H_2_S levels and H2S synthesis in streptozotocin induced Type-2 diabetes-an experimental study based on Swietenia macrophylla seeds. Asian Pac J Trop Biomed.

[43] Yusuf M (2005). Streptozotocin-induced diabetes in the rat is associated with enhanced tissue hydrogen sulfide biosynthesis. Biochem Biophys Res Commun.

[44] Muniyappa R, Sowers JR (2013). Role of insulin resistance in endothelial dysfunction. Rev Endocr Metab Disord.

[45] Giannarelli R, Aragona M, Coppelli A, Del Prato S (2003). Reducing insulin resistance with metformin: the evidence today. Diabetes Metab.

[46] Fuhita Y, Inagaki N (2017). Metformin: new preparations and nonglycemic benefits. Curr Diab Rep.

[47] Shah Z (2011). Acute DPP-4 inhibition modulates vascular tone through GLP-1 independent pathways. Vascul Pharmacol.

[48] Sicras-Mainar A, Navarro-Artieda R (2014). Use of metformin and vildagliptin for treatment of type 2 diabetes in the elderly. Drug Des Devel Ther.

[49] Apaijai N, Chinda K, Palee S, Chattipakorn S, Chattipakorn N (2014). Combined vildagliptin and metformin exert better cardioprotection than monotherapy against ischemia-reperfusion injury in obese-insulin resistant rats. PLoS One.

[50] Carolo dos Santos K (2014). Cardiac energy metabolism and oxidative stress biomarkers in diabetic rat treated with resveratrol. PLoS One.

[51] Katakam PV, Ujhelyi MR, Hoenig M, Miller AW (2000). Metformin improves vascular function in insulin-resistant rats. Hypertension.

[52] Kosaraju J (2013). Vildagliptin: an anti-diabetes agent ameliorates cognitive deficits and pathology observed in streptozotocin-induced Alzheimer’s disease. J Pharm Pharmacol.

[53] Lim AK (2011). Evaluation of JNK blockade as an early intervention treatment for type 1 diabetic nephropathy in hypertensive rats. Am J Nephrol.

[54] Buikema H (2000). Comparison of zofenopril and lisinopril to study the role of the sulfhydryl-group in improvement of endothelial dysfunction with ACE-inhibitors in experimental heart failure. Br J Pharmacol.

[55] Gschwend S (2002). Endothelial dilatory function predicts individual susceptibility to renal damage in the 5/6 nephrectomized rat. J Am Soc Nephrol.

